# Supplementary material for: From sequence to enzyme mechanism using multi-label machine learning
Source: BMC Bioinformatics. 2014 May 19;15:150. doi: 10.1186/1471-2105-15-150 (PMC4229970; doi:10.1186/1471-2105-15-150)
Supplement: Additional file 2 — Java code of ml2db. Additional file ml2db_code.tar.gz contains the Java source code to run the multi-label machine learning experiments and save the results to database. The code’s Javadoc is included. [file 1471-2105-15-150-S2.zip › additional file 2/ml2db/ml2db/doc/main/ml2db/weka/IdentifiedSparseInstance.html]

IdentifiedSparseInstance


JavaScript is disabled on your browser.


- Overview
- Package
- Class
- Use
- Tree
- Deprecated
- Index
- Help

- Prev Class
- Next Class

- Frames
- No Frames

- All Classes

- Summary:
- Nested |
- Field |
- Constr |
- Method

- Detail:
- Field |
- Constr |
- Method


main.ml2db.weka

## Class IdentifiedSparseInstance

- java.lang.Object
- - weka.core.AbstractInstance
  - - weka.core.SparseInstance
    - - main.ml2db.weka.IdentifiedSparseInstance

- All Implemented Interfaces:
  :   java.io.Serializable, weka.core.Copyable, weka.core.Instance, weka.core.RevisionHandler

  ---

    

  ```
  public class IdentifiedSparseInstance
  extends weka.core.SparseInstance
  ```

  Class

  Version:
  :   23 Jun 2011

  Author:
  :   Luna De Ferrari ldf(at)st-andrews.ac.uk

  See Also:
  :   Serialized Form

- - ### Constructor Summary

    Constructors

    Constructor and Description

    `IdentifiedSparseInstance()`
  - ### Method Summary

    - ### Methods inherited from class weka.core.SparseInstance

      `copy, getRevision, index, locateIndex, main, mergeInstance, numAttributes, numValues, replaceMissingValues, setValue, setValueSparse, toDoubleArray, toStringNoWeight, value`
    - ### Methods inherited from class weka.core.AbstractInstance

      `attribute, attributeSparse, classAttribute, classIndex, classIsMissing, classValue, dataset, deleteAttributeAt, enumerateAttributes, equalHeaders, equalHeadersMsg, hasMissingValue, insertAttributeAt, isMissing, isMissing, isMissingSparse, numClasses, relationalValue, relationalValue, setClassMissing, setClassValue, setClassValue, setDataset, setMissing, setMissing, setValue, setValue, setValue, setWeight, stringValue, stringValue, toString, toString, toString, value, valueSparse, weight`
    - ### Methods inherited from class java.lang.Object

      `equals, getClass, hashCode, notify, notifyAll, wait, wait, wait`

- - ### Constructor Detail


    - #### IdentifiedSparseInstance

      ```
      public IdentifiedSparseInstance()
      ```


- Overview
- Package
- Class
- Use
- Tree
- Deprecated
- Index
- Help

- Prev Class
- Next Class

- Frames
- No Frames

- All Classes

- Summary:
- Nested |
- Field |
- Constr |
- Method

- Detail:
- Field |
- Constr |
- Method
